# Delirium and subsyndromal delirium are associated with the long-term risk of death after ischaemic stroke

**DOI:** 10.1007/s40520-021-02071-y

**Published:** 2022-01-11

**Authors:** Elzbieta Klimiec-Moskal, Agnieszka Slowik, Tomasz Dziedzic

**Affiliations:** Department of Neurology, Jagiellonian University Medical College, ul. Botaniczna 3, 31-503 Kraków, Poland

**Keywords:** Delirium, Subsyndromal delirium, Mortality, Stroke

## Abstract

**Background:**

Post-stroke delirium has a negative impact on functional outcome. We explored if there is any association between delirium, subsyndromal delirium and long-term mortality after ischaemic stroke and transient ischaemic attack.

**Methods:**

We included 564 patients with ischaemic stroke or transient ischaemic attack. We assessed symptoms of delirium during the first 7 days after admission. We used Cox proportional hazards models to analyse all-cause mortality during the first 5 years after stroke.

**Results:**

We diagnosed delirium in 23.4% and subsyndromal delirium in 10.3% of patients. During the follow-up, 72.7% of patients with delirium, 51.7% of patients with subsyndromal delirium and 22.7% of patients without delirious symptoms died (*P* < 0.001). Patients with subsyndromal delirium and delirium had higher risk of death in the multivariate analysis (HR 1.72, 95% CI 1.11–2.68, *P* = 0.016 and HR 3.30, 95% CI 2.29–4.76, *P* < 0.001, respectively).

**Conclusions:**

Post-stroke delirium is associated with long-term mortality. Patients with subsyndromal delirium are at the intermediate risk of death.

## Introduction

Delirium is a common complication after stroke affecting between 10 and 48% of patients [1]. Despite the transient nature of symptoms, delirium is associated with poor functional outcome [2]. Stroke patients with delirium have increased risk of death up to 1 year [2, 3]. The association between delirium and longer-term mortality was observed in non-stroke population [4, 5]. Only one retrospective study investigated stroke patients showing no independent association between delirium and long-term mortality [6]. Therefore, the relationship between post-stroke delirium and long-term mortality remains unclear.

Subsyndromal delirium (SSD) refers to patients who manifest delirious symptoms but do not meet diagnostic criteria for delirium [7]. SSD is a relatively new concept, and its clinical significance is yet to be established. The risk of poor functional outcome in SSD patients is increased and intermediate between patients with delirium and those without delirious symptoms [7, 8]. The association between SSD and the risk of death is less clear and seems to vary across patients with different underlying conditions [7, 9, 10]. Two studies on stroke patients have not found any association between SSD and 3-month and 1-year mortality [8, 11]. However, there is a lack of study with a longer follow-up.

We explored if there is any association between delirium, SSD and the 5-year risk of death in ischaemic stroke patients.

## Methods

We recruited patients among participants of the Prospective Observational Polish Study on Delirium (PROPOLIS). The PROPOLIS was conducted between May 2014 and March 2016 in the Department of Neurology, University Hospital, Krakow, Poland. The study protocol was approved by the Bioethics Committee of the Jagiellonian University and is published elsewhere [12]. Informed consent was obtained from participants or their caregivers.

The methodology for the current study was previously described [8]. Briefly, the inclusion criteria were as follows: (1) ischaemic stroke or transient ischaemic attack; (2) age ≥ 18 years; (3) admission to hospital within 48 h after symptoms onset; and (4) Polish as a native language. We excluded patients in whom we were not able to use the confusion assessment method (CAM) due to their neurological deficit.

We examined the symptoms of delirium daily during the first 7 days of hospitalization. To assess core delirium symptoms, we used the Brief CAM for verbal patients [13] and the Intensive Care Units CAM for non-verbal patients [14]. We diagnosed delirium using the Diagnostic and Statistical Manual of Mental Disorders, 5th edition (DSM-5) criteria [15]. Next, we assigned each patient to one of three groups: delirium, SSD or no delirium/no subsyndromal delirium (ND/NSSD). We defined SSD as the presence of one or more core symptoms of delirium that did not meet the DSM-5 criteria and did not progress to the full syndrome. We included patients without any core feature of delirium in ND/NSSD group. We used the National Institute of Health Stroke Scale (NIHSS) to examine the neurological deficit; the modified Rankin Scale to assess pre-stroke dependency; and the Informant Questionnaire on Cognitive Decline in the Elderly to assess the pre-morbid cognitive impairment [8, 12]. We obtained data on all-cause 5-year mortality from the government-maintained database.

We used Kaplan–Meier curves and the log-rank test to compare survival among groups. Then, we used Cox proportional hazards models to identify independent predictors of death. We adjusted the multivariate analysis for clinical predictors, which were significant in the univariate analysis (*P* < 0.050). To impute missing data on pre-stroke cognitive impairment, we implemented the multiple imputation method using 10 imputations. We performed statistical analyses in STATA version 16 (StataCorp, College Station, TX).

## Results

We included 564 patients with ischaemic stroke or transient ischaemic attack who underwent CAM assessment during the first 7 days of hospitalization. We assigned 58 patients (10.3%) to SSD group, 132 (23.4%) to delirium group and 374 (66.3%) to ND/NSSD group.

Detailed characteristics of our cohort were published elsewhere [8]. Briefly, SSD and delirium patients were older, had more severe neurological deficit on admission and more often suffered from atrial fibrillation and diabetes mellitus, pre-stroke cognitive impairment and dependency than ND/NSSD patients.

**Fig. 1:**
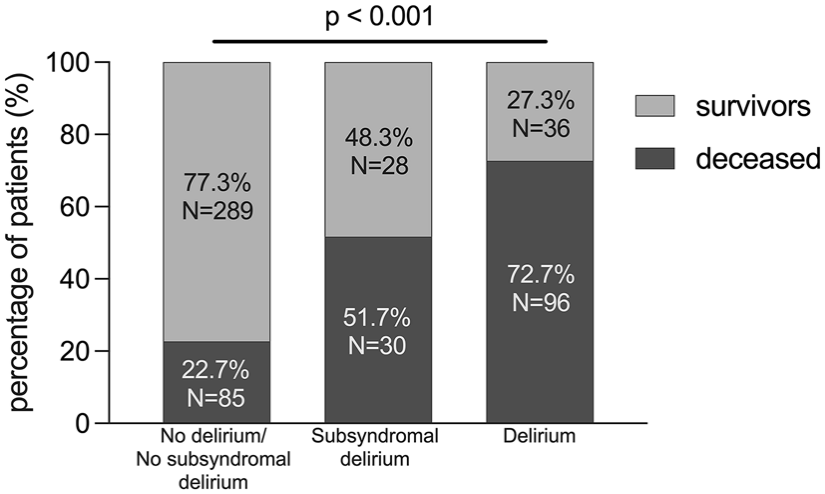
Five-year mortality rate in three groups

**Fig. 2:**
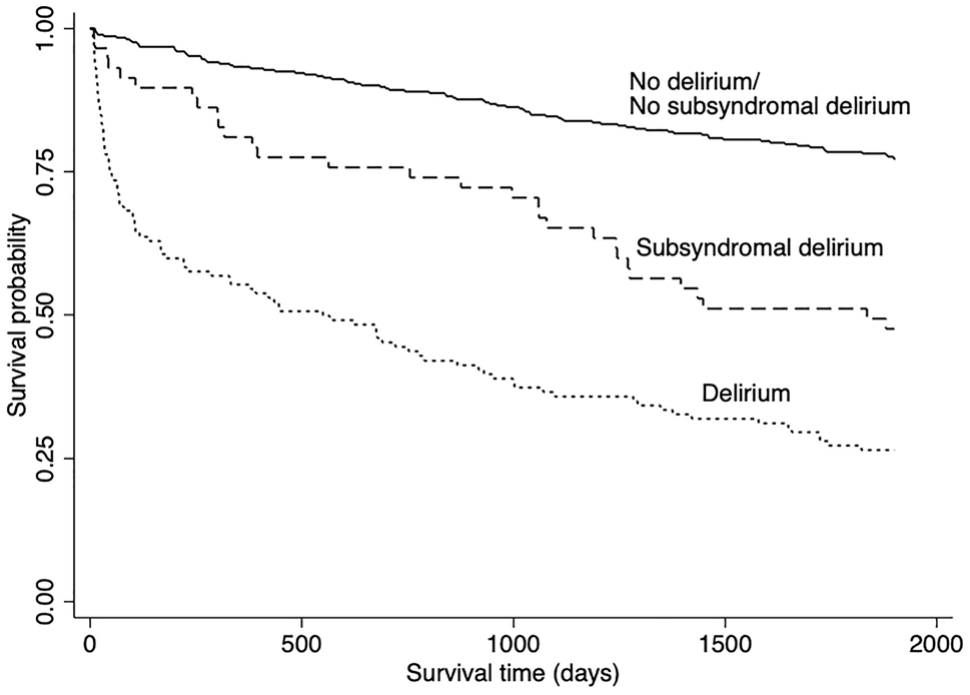
Kaplan–Meier curves for three groups

Overall, 211 patients (37.4%) patients died during the first 5 years after stroke (Fig. 1). Kaplan–Meier curves are shown in Fig. 2. The log-rank test showed significant differences in survival among 3 groups (*P* < 0.001).

In the univariate analysis, SSD patients (HR 2.82, 95% CI 1.86–4.29, *P* < 0.001) and delirium patients (HR 5.66, 95% CI 4.21–7.61, *P* < 0.001) had the increased risk of death compared with ND/NSSD group. Delirium patients also had the increased risk of death compared with SSD patients (HR 2.01, 95% CI 1.34–3.04, *P* = 0.001).

Multivariate analysis was adjusted for predictors identified in the univariate analysis: age (HR 1.08, 95% CI 1.07–1.10, *P* < 0.001), atrial fibrillation (HR 2.67, 95% CI 2.01–3.55, *P* < 0.001), diabetes mellitus (HR 1.36, 95% CI 1.02–1.81, *P* = 0.034), myocardial infarction (HR 1.54, 95% CI 1.08–2.18, *P* = 0.015), pre-stroke cognitive impairment (HR 2.28, 95% CI 1.63–3.18, *P* < 0.001), pre-stroke dependency (HR 3.70, 95% CI 2.64–5.17, *P* < 0.001), NIHSS score on admission (HR 1.06, 95% CI 1.04–1.09, *P* < 0.001) and lesion location in the right hemisphere (HR 1.65, 95% CI 1.26–2.16, *P* < 0.001). Data on pre-stroke cognitive impairment were imputed for 101 patients for whom we have no information about pre-stroke cognitive status. In the multivariate analysis, the risk of death was 1.72 times higher in SSD group (95% CI 1.11–2.68, *P* = 0.016) and 3.30 times higher in delirium group (95% CI 2.29–4.76, *P* < 0.001) compared with ND/NSSD group. Delirium patients also had the increased risk of death (HR 1.65, 95% CI 1.05–2.60, *P* = 0.029) compared to SSD patients.

## Discussion

Our study demonstrated that both delirium and SSD are independent predictors of 5-year mortality after stroke. The risk of death in SSD patients was intermediate between patients with delirium and those without any delirious symptom.

Only one study investigated the association between delirium and risk of death beyond 1 year after stroke. It showed higher 10-year mortality in patients with post-stroke delirium, but this association did not withstand adjustment for potential confounders [6]. Some limitations of that study merit consideration. Authors made the diagnosis of delirium retrospectively, which might bring a risk of patient misclassification. In addition, stroke itself increases the risk of death and can mask the effect of delirium [4]. A sample size of that study (50 delirious patients) might be too small to demonstrate the additional effect of delirium on long-term mortality.

Our findings are in accordance with the results obtained from patients undergoing cardiac surgery, which showed the association between delirium and long-term mortality [4, 5]. The association between delirium and worse outcomes, however, is not fully explained. Delirium may be a sign of pre-existing pathology that together with superimposed stressors such as stroke is related to worse outcome [16]. On the other hand, delirium itself may imply pathological processes that cause persistent cognitive dysfunction and worsened mental health, which, in turn, could disturb recovery after stroke [1, 16]. Impaired recovery, leading to decline in functional status and to functional dependency, may be an important factor contributing to long-term mortality.

In our study, not only delirium, but also SSD was related to long-term mortality after stroke. Previous studies did not find the link between SSD and 3-month [11] and 1-year mortality [8]. Possibly, SSD may be a marker of the underlying conditions (e.g. cognitive impairment, frailty), which are not severe enough to contribute significantly to short-term mortality, but their effect cumulate over time. This association may also depend on investigated population. SSD has a limited impact on patients hospitalized in intensive care units. In this group, other factors (e.g. severe comorbidities) are stronger predictors of outcome. Moreover, these patients are more likely to develop full syndrome [9]. In contrast, in non-intensive care patients (e.g. surgical or palliative) SSD was shown to be associated with greater mortality [7].

Our results highlight the need for assessment of delirious symptoms in the acute phase of stroke to identify patients who require long-term monitoring and medical care. Furthermore, SSD is thought to represent a subthreshold state of delirium. However, in contrast to delirium, different studies yielded inconsistent results on an association between SSD and outcome [9, 11]. Our current and previous study [8], demonstrating an intermediate risk of death and poor functional outcome in SSD patients, support an idea of SSD as a separate entity with clinical significance.

Our study has several limitations. First, assessment of delirious symptoms was limited to the first week after stroke, what might result in misclassification of patients with delayed delirium symptoms. Second, we analysed only all-cause mortality. We were not able to investigate whether delirium or SSD is associated with any specific cause of death. Third, we did not analyse delirious symptoms separately, while some of them such as acute-onset or disorganized thinking may be stronger predictors of mortality than others [10]. Finally, there is a lack of accepted definition of SSD and various criteria were previously used [17]. SSD can be defined categorically using dichotomous presence of one or two core diagnostic features, or dimensionally based on predefined severity score. However, criterium of one feature present may be overinclusive. The approach incorporating both categorical and dimensional elements is recommended in the future studies.

In conclusion, our results suggest that delirium and, to a lesser degree, SSD are associated with the increased long-term risk of death after stroke.

## Data Availability

The data that support the findings of this study are available from the corresponding author upon reasonable request.
